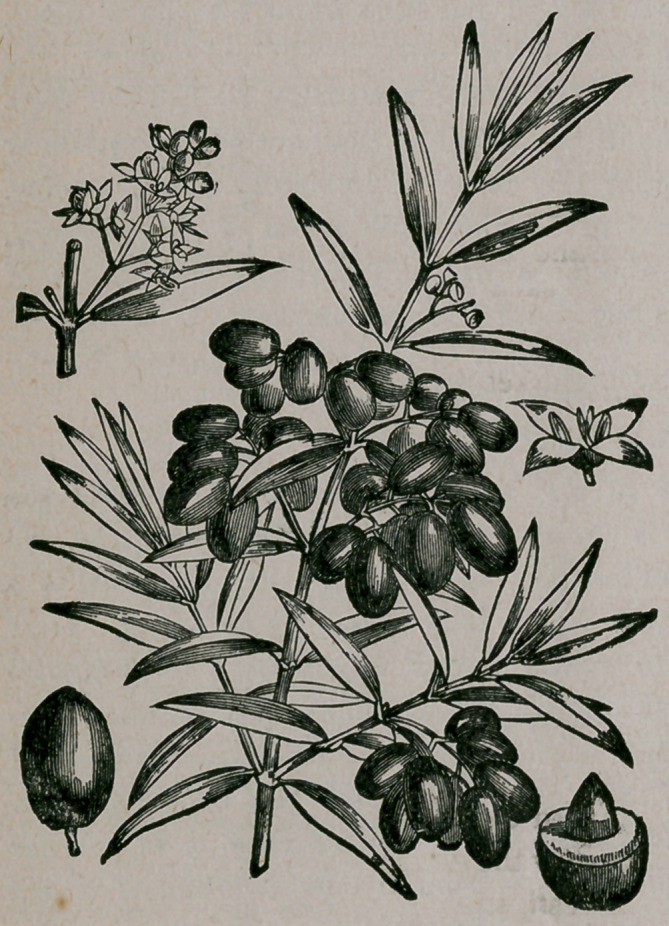# The Olive Tree

**Published:** 1887-11

**Authors:** 


### THE OLIVE TREE.

The olive tree, *Olea Europa*, grows abundantly in all the countries bordering on the Mediterranean Sea. It thrives upon the most rocky calcareous soils, seldom exceeds twenty feet in height, but is much branched and spreading; it lives to a great age, and increases very much in bulk, so that one tree may easily, at a little distance, be mistaken for a group. There is an olive tree at Pescio seven hundred years old, and twenty-five feet in circumference. The trees also grow abundantly in Judea, and there are some still standing in the garden of Gethsemane, which are so large and old, that they are thought to have been in existence ever since the time of Jesus. Josephus tells us that when Titus destroyed Jerusalem, he cut down all the trees within one hundred furlongs of the city; still it is very probable that these trees may have grown up from the roots of the old ones, because it is quite a characteristic of the olive-tree to shoot up again, however frequently it may be cut down. They are wild olives of extreme old age, and their stems quite rough and gnarled. The leaves of the tree are evergreen, stiffish and pointed; the flowers white, growing in clusters, succeeded by an oval *drupe*, or plum, which is violet-colored when ripe, bitter and nauseous. The preserved olives, common as a table luxury, are the unripe fruit pickled in a strong solution of salt. Salad oil is made from olives.

The ripe fruit is gathered in November, and bruised in a mill, the stones of which are set so wide apart as not to bruise the nut or kernel; the pulp is then gently pressed in bags made of rushes; the first oil that flows is of the most value, a second quality is obtained by breaking the refuse, mixing it with warm water, and returning it to the press; and after this a third very inferior kind is obtained.

It is found by experiment that olive trees grow as thriftily along the coast line of California, all the way from San Louis Obispo to San Diego, as in their native clime, on the borders of the Mediterranean, and other Asiatic regions. Col. Edward Cooper of Santa Barbara, is even said to be the largest grower of olives, and manufacturer of sweet oil, in the world. His orchard of one hundred acres, of which eighty acres of trees are in bearing, is in a most prosperous condition. In a recent conversation in relation to this enterprise, he is reported to have said, "I began in rather a small way in 1873, and have planted from one thousand to three thousand trees each winter ever since. It takes the trees from four to six or seven years to bear. I expect to make from my present crop from twenty thousand to twenty-five thousand bottles of oil. There is a market right here at home for every bottle that can be made. California cannot produce the tenth part that is required. The present crop is very good. The trees are in fine condition, and are bearing well. I employ thirty men steadily, and a good deal of the time I have from sixty to seventy."

This being the case, there is a prospect that our grocers will not always be driven to the extremity of palming upon their customers, cotton seed oil and other imitations in elegant French labels.

## Figures and Tables

**Figure f1:**